# The spatiotemporal regulations of epicatechin biosynthesis under normal flowering and the continuous inflorescence removal treatment in *Fagopyrum dibotrys*

**DOI:** 10.1186/s12870-022-03761-z

**Published:** 2022-07-29

**Authors:** Xinwei Guo, Zuliang Luo, Min Zhang, Linfang Huang, Hui Wang, Yuting Li, Xu Qiao, Ailian Li, Bin Wu

**Affiliations:** 1 Key Laboratory of Bioactive Substances and Resources Utilization of Chinese Herbal Medicine, Ministry of Education and National Engineering Laboratory for Breeding of Endangered Medicinal Materials, Institute of Medicinal Plant Development, Chinese Academy of Medical Sciences and Peking Union Medical College, Beijing, 100193 China; 2 Institute of Sericulture, Chengde Medical University, Chengde, 067000 China

**Keywords:** Flowering, Continuous inflorescence removal, Epicatechin, *Fagopyrum dibotrys*, Rhizome/root plant

## Abstract

**Background:**

Flowering is a critical physiological change that interferes with not only biomass yield but also secondary metabolism, such as the biosynthesis of flavonoids, in rhizome/root plants. The continuous inflorescence removal (CIR) treatment is frequently conducted to weaken this effect. *Fagopyrum dibotrys* (D.Don) H.Hara (Golden buckwheat) is a kind of rhizome medicinal plant rich in flavonoids and is widely used for the treatment of lung diseases. The CIR treatment is usually conducted in *F. dibotrys* because of its excessive reproductive growth. To uncover the molecular mechanisms, comprehensive analysis was performed using metabolome and transcriptome data obtained from normally bloomed and the CIR treated plants.

**Results:**

Metabolome results demonstrated that in the rhizomes of *F. dibotrys*, its bioactive compound called epicatechin has higher amount than most of the detected precursors. Compared with the normally bloomed plants, the level of epicatechin in the rhizomes of the CIR group increased by 25% at the withering stage. Based on 96 samples of the control and the CIR groups at 4 flowering stages for 4 tissues, RNA-Seq results revealed a 3 ~ 5 times upregulations of all the key enzyme genes involved in the biosynthesis of epicatechin in both time (from the bud stage to the withering stage) and spatial dimensions (from the top of branch to rhizome) under the CIR treatment compared to normal flowering. Integrated analysis of LC–MS/MS and transcriptome revealed the key roles of several key enzyme genes besides anthocyanidin reductase (*ANR*). A total of 93 transcription factors were identified to co-expressed with the genes in epicatechin biosynthetic pathway. The flowering activator SQUAMOSA promoter-binding protein like (*SPLs*) exhibited opposite spatiotemporal expression patterns to that of the epicatechin pathway genes; *SPL3* could significantly co-express with all the key enzyme genes rather than the flowering repressor *DELLA*. Weighted gene co-expression network analysis (WGCNA) further confirmed the correlations among chalcone synthases (*CHSs*), chalcone isomerases (*CHIs*), *ANRs*, *SPLs* and other transcription factors.

**Conclusions:**

*SPL3* might dominantly mediate the effect of normal flowering and the CIR treatment on the biosynthesis of epicatechin in rhizomes mainly through the negative regulations of its key enzyme genes including *CHS*, *CHI* and *ANR*.

**Supplementary Information:**

The online version contains supplementary material available at 10.1186/s12870-022-03761-z.

## Background

Flowering is a critical physiological change during reproductive growth that interferes with not only biomass yield but also secondary metabolism. Generally, the process of flowering is divided into 4 stages: the bud stage, the initial flowering stage, the flowering stage and the withering stage [1]. In *Arabidopsis*, numerous interwoven genetic pathways exemplified by vernalization, photoperiod/circadian clock, gibberellin, autonomous, and aging could regulate flowering process [2]. On physiological level, the above pathways can end at promotion of the flowering process by repression of vegetative growth, or by up-regulation of reproductive growth [3]. The transcription factors (TFs) called SQUAMOSA PROMOTER-BINDING PROTEIN-LIKEs (*SPLs*), which is known as plant “aging genes”, has been proved to play a dominate role during the whole process of vegetative-to-reproductive transition and flowering: as the plant gets mature and progresses toward flowering, the expressions of *SPLs* are programmed to increase till the withering stage [4]. The repressor of gibberellin signaling pathway called *DELLA* could interferes with *SPL* transcriptional activity and consequently delays floral transition [5].

Recently, vegetative and reproductive growth were found to have different effects on the biosynthesis of secondary metabolites in plants. The accumulation rate of many bioactive compounds decreased dramatically in roots when the plants stepped into reproductive stage [6, 7]. For instance, the biosynthesis of anthocyanin, one of the products of flavonoid pathway, could be improved by vegetative growth but repressed by reproductive growth through the interaction network involving *SPLs*, *DELLAs*, *ABIs*, dihydroflavonol 4-reductases (*DFRs*) or MYBs [8–12]. The abundance of *SPL9/10/13* in the inflorescences could repress the accumulation of anthocyanin by directly down-regulating its key enzyme genes. Notably, for the *Arabidopsis* plants at flowering stage, anthocyanin has been found to accumulate in an acropetal manner with the highest level at the junction between rosette and stem, and the lowest level at the inflorescence top, and this pattern was regulated by *SPL* genes through the opposite spatial expression patterns; the high levels of *SPL9* in the inflorescences repress anthocyanin accumulation by directly preventing expression of anthocyanin biosynthetic genes, such as anthocyanidin synthase (*ANS*), flavonoid 3’-hydroxylase (*F3’H*), *DFR* and *UGT75C1*, through destabilization of the MYB-bHLH-WD40 transcriptional activation complex [12]. Therefore, it is indicated that spatially, the repressing gradient of the genes in flavonoid pathway by the differential expressions of *SPLs* might be one of the important molecular mechanisms for the effects of flowering on secondary metabolism in plant.

In root/rhizome medicinal plants, especially for the perennial herbs belonging to *Polygonaceae*, *Asteraceae* or *Apiaceae*, the genus with a large number of branched stems and inflorescences, the treatment of inflorescence removal/continuous inflorescence removal (CIR) is usually applied to weaken the negative effects of reproductive growth on the yield and quality of roots/rhizomes [13, 14]. It was proved that these treatments have great effects on the biosynthesis of some secondary metabolites. In *Bupleurum chinense* and *Panax notoginseng* plants, these treatments significantly improved the biosynthesis of triterpenoid in the roots/rhizomes by activating the metabolomic acid (MVA) and methylerythritol phosphate (MEP) pathways, but decrease the accumulation of flavonoids in *P. notoginseng* [13, 15]. These studies indicated that the regulation of normal flowering or the CIR treatment on the secondary metabolism in rhizomes/roots might depend on metabolites or plant species. However, the underlying molecular mechanism have been poorly studied.

*Fagopyrum dibotrys* (D. Don) H.Hara (Golden buckwheat) is one of important medicinal plants which mainly distributed in China, India, Thailand and Vietnam [16]. Its dried rhizomes which called Fagopyri Dibotryis Rhizoma is an important crude drug and functional food that widely used for the treatment of lung disease, dysentery, rheumatism and throat inflammation [17]. Flavonoids have been shown to be the major active ingredients in *F. dibotrys*. The content of flavonoids in *F. dibotrys* is higher than that found in other buckwheat cultivars [18]. As a member of the genus *Polygonaceae*, one of the biggest problems in the cultivation of *F. dibotrys* is the excessive reproductive growth due to the large number of branched stems and inflorescences [19]. The biomass of the reproductive organs account for approximately 30% of the whole plant [20]. Moreover, its bioactive compound called epicatechin, one of the products of flavonoid pathway, could also be synthesized in non-medicinal organs such as flowers or leaves, resulting in serious competition in the biosynthesis of epicatechin between aboveground organs and rhizome [21]. To enhance the biosynthesis of epicatechin in rhizomes, the CIR treatment is usually conducted in *F. dibotrys* but with unclear molecular mechanism. Thus, we believe that *F. dibotrys* could be used as a model rhizome medicinal plant to investigate the molecular regulations of normal flowering and the CIR treatment on the biosynthesis of epicatechin or other flavonoids.

To date, it has been revealed that the biosynthesis of epicatechin is via the phenylpropanoid and the flavonoid pathway; several key enzyme genes have been cloned, including phenylalanine ammonia-lyase (*PAL*), flavonol synthase (*FLS*), chalcone isomerase (*CHI*), chalcone synthase (*CHS*), *DFR*, *ANS*, leucoanthocyanidin reductase (*LAR*), etc. [22–24]. Anthocyanidin reductase (*ANR*) was proved to be the key enzyme gene of the biosynthesis of epicatechin in *F. dibotrys* [17].

In this study, we aimed to investigate the spatiotemporal regulations of the biosynthesis of epicatechin by normal flowering and the CIR treatment in *F. dibotry*. Metabolomics and transcriptome were performed, and comprehensively analyzed. All the results would provide new insight into the molecular mechanism of normal flowering- or the CIR-regulated biosynthesis of epicatechin or other flavonoids in rhizome/root medicinal plants.

## Results

### The epicatechin level in rhizome under normal flowering and the CIR treatment

The line of *F. dibotry* used in this study was bred by us previously [19]. Under normal growth condition, the amounts of epicatechin and its precursors in the harvested rhizomes were detected and quantified by metabolomic analysis. As a result, a total of five precursors were identified, including L-phenylalanine (the amount, the same below 5.71 × 10^6^ ± 3.35 × 10^6^), cinnamate (6.92 × 10^3^ ± 3.15 × 10^3^), narigenin ((9.37 × 10^4^ ± 4.40 × 10^4^), eriodictyol (6.94 × 10^4^ ± 3.10 × 10^4^) and flavanone ((2.27 × 10^4^ ± 9.54 × 10^3^). The amount of epicatechin was (5.02 × 10^6^ ± 1.34 × 10^6^), which has no significant difference with that of L-phenylalanine, but was dozens or hundreds of times higher than that of the most detected precursors (Supplementary Table S2).

**Fig. 1:**
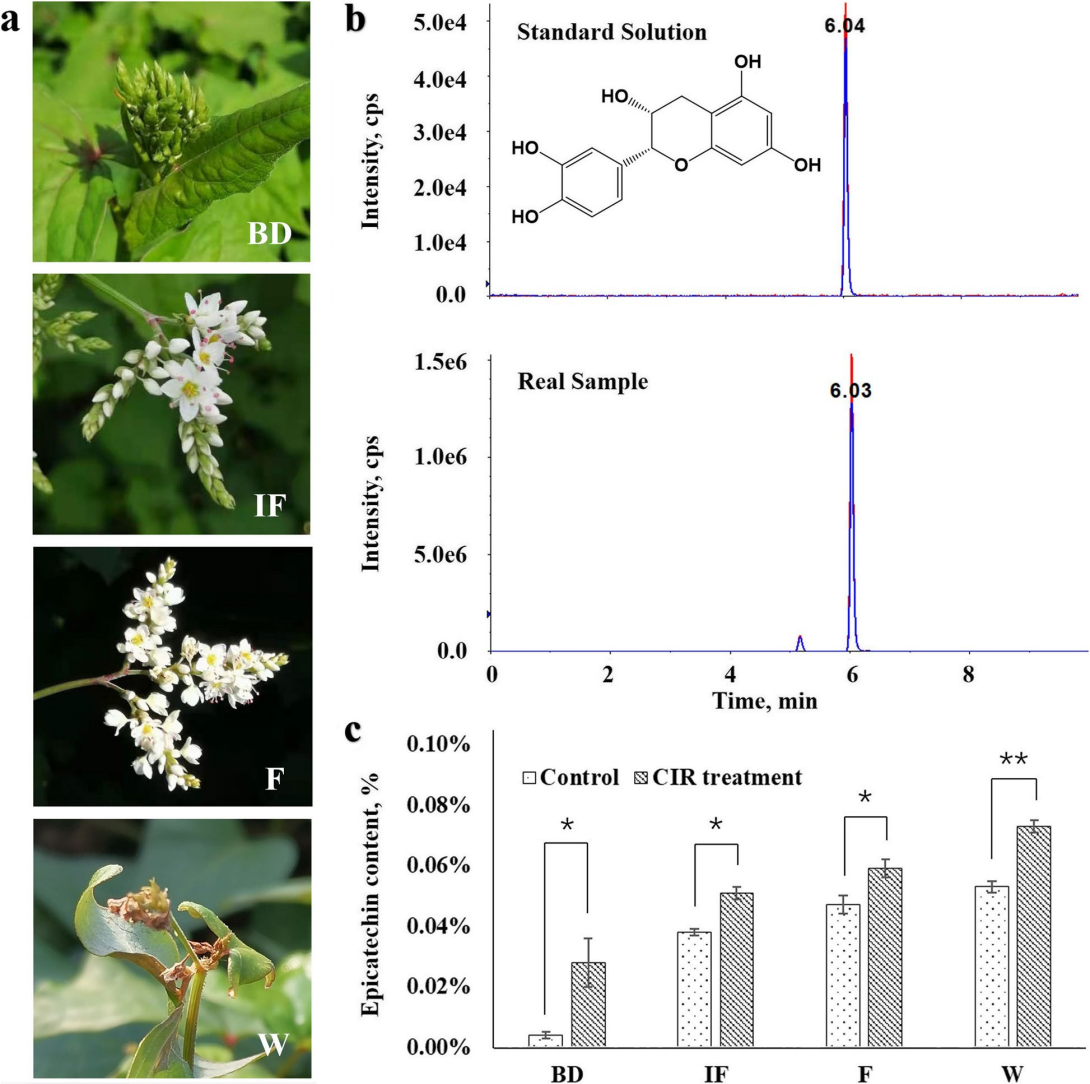
The epicatechin levels in *F. dibotrys* under normal flowering and the CIR treatment. **a** The morphologic characteristics of floral organs in the 4 sampling stages: BD, the bud stage, when most buds appear and are still green; IF, the initial flowering stage, when most buds are preparing to bloom (turns to white); F, the full flowering stage, when most buds bloom completely; W, the withering stage, when all the flowers withered and the rhizome was harvested. **b** Chemical structures and typical LC–MS/MS total ion chromatograms of epicatechin, including the standard solutions of the reference substance of epicatechin (the top) and the rhizome samples (the bottom). **c** The changes on epicatechin levels in the rhizomes under normal flowering and the CIR treatment at the 4 stages, each sample has 3 biological replications

The morphologic characteristics of floral organs at the 4 sampling stages were shown in Fig. 1a. A total of 75 days of treatment were conducted after the appearance of floral bud. The levels of epicatechin in the dried rhizomes were detected by LC–MS/MS and the extracted ion chromatograms of the reference substance of epicatechin and the samples were shown in Fig. 1b. As expected, the average content of epicatechin in the CIR group was significantly higher than that of the normal flowering group in each stage, and finally reached to 0.73 ± 0.002%, which improved by 25% in comparison with the normally bloomed plants (Fig. 1c).

At the bud stage (BD), the difference between the two groups reached to the maximum of 7 times. We speculated the reason was: the CIR treatment started at Aug 21 when the buds firstly appeared; while the sampling time of the bud stage was 7 days after inflorescence appearance (Aug 28) when most of the buds appeared. Thus, this one-week CIR treatment might be critical for the accumulation of epicatechin in rhizomes. During the following 3 stages, the increasement of epicatechin slowed down in the normal flowering group, but kept increasing till the withering stage (W) in the CIR group, indicating the great effects of the CIR treatment on the biosynthesis of epicatechin in the rhizomes of *F. dibotry*.

### RNA Sequencing, assembly and annotation

To explore the spatiotemporal regulation of the biosynthesis of epicatechin by normal flowering and the CIR treatment in *F. dibotrys*, 96 RNA-Seq libraries (2 groups, 4 stages, 4 different tissues, 3 biological replicates) were constructed. After sequencing, a total of 20.13 ~ 29.96 million raw reads were generated. After removing the reads that contained adapter or poly-N and the low quality, 19.75 ~ 28.32 million clean reads were obtained, with the GC contents ranging from 43.54% to 46.69%, respectively (Supplementary Table S3). A total of 76,952 unigenes were assembled with the lengths of 301 ~ 20,556 bp, and the mean value was 1377 bp (Supplementary Table S4). Finally, 55.82%, 38.25%, 23.03%, 45.41%, 43.96%, 43.96% and 17.64% of the assembled unigenes were annotated by the Nr, Nt, KO, SwissProt, Pfam, GO and KOG databases, respectively (Supplementary Table S5).

### PCA and cluster analysis of the relationships among the sequencing samples

**Fig. 2:**
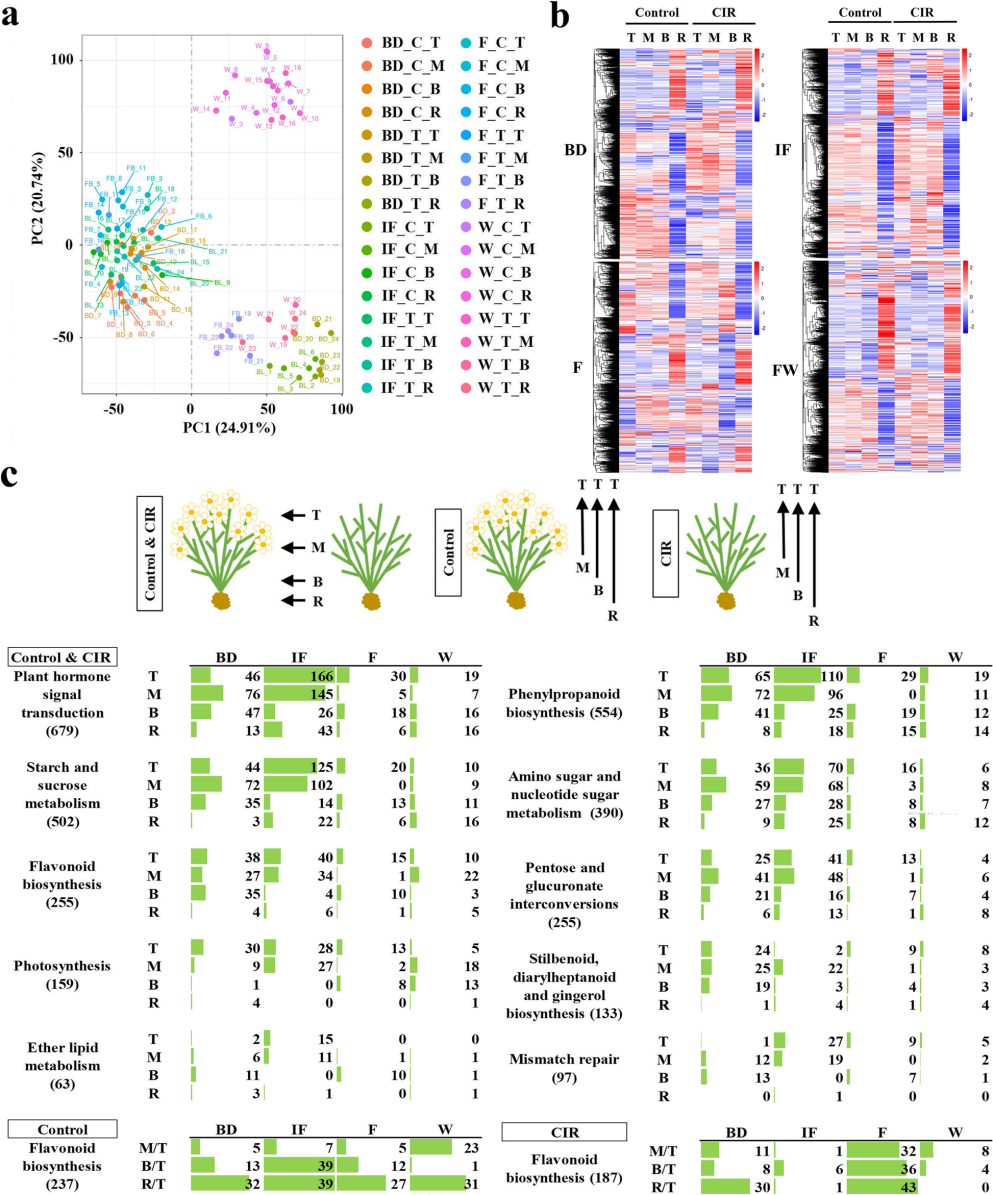
The bioinformatic analysis of the transcriptome data of normal flowering and the CIR treatment. **a** The PCA analysis of the expression patterns of all the 96 samples at the 4 stages. In the legend, the first character indicated the name of the stage, which was same with Fig. 1a; the second character indicated the name of the group, the normal flowering group and the CIR group were indicated as C and T, respectively; the last character indicated the 4 types of tissue: T, the top of the branch; M, the middle part of the branch, at 1/2 of plant height; B, the basal part of the branch; R, the rhizome of each plant. Three biological replications were conducted. **b** the H-cluster analysis of the expression patterns of all the 96 samples at the 4 stages. The abbreviations were same as that of Fig. 2a. **c** The KEGG enrichment analysis. The schematic diagram in the top pattern indicated the different ways of KEGG analysis, which included the comparison between the groups of normal flowering and the CIR treatment, and the comparison within each group. In the schematic diagram, the direction of the arrow indicated the control samples. The numeric characters indicated the number of DEGs

PCA analysis was performed to study the relationships among the 32 *F. dibotrys* samples (after taking the average coverage of the three biological replicates). As shown in Fig. 2a, all the samples were grouped into three clusters: the first group was the branch samples from the first 3 stages (BD, IF, F); the second was the branch samples from the W stage; the third was all the rhizome samples, which indicated the significant difference between branch and rhizome samples. The H-cluster analysis further showed that the overall expression profiles of rhizome samples were opposite to that of the branch samples; and the global expression levels in the rhizome samples were apparently higher or lower than that of the branch samples, indicating the higher sensitivity of rhizome to flowering or the CIR treatment (Fig. 2b).

### GO and KEGG enrichment of DEGs

Using the threshold of the adjusted *P* value < 0.05 and |log_2_FoldChange|> 1, a total of 614 ~ 8,222 DEGs were finally identified in each comparison, based on the average coverage of three biological replicates (Supplementary Table S6). In comparison with the normal flowering group, GO enrichment analysis showed that the most abundant DEGs in the CIR group were identified in “biological process” and the “secondary metabolic process” pathways (Supplementary Fig. S1).

Taken the group of normal flowering as the control, the top 10 KEGG pathways with the most significant enrichment DEGs in the CIR groups were assigned in plant hormone signal transduction, phenylpropanoid biosynthesis, metabolism of sucrose and sugar, etc., flavonoid biosynthesis was ranked the fifth (Fig. 2c, Control & CIR). Moreover, the DEG numbers from the top 10 KEGG pathways gradually decreased from the top of the branch to the rhizome, which indicated that the closer to the rhizome, the more similar for the genes that being responsive to normal flowering and the CIR treatment.

Within the group of normal flowering, when taking the sample T (the top part of branch) as the control, the number of DEGs in flavonoid pathway increased from the top of the branch to the rhizome (Fig. 2c, the Control group), which confirmed the higher sensitivity of rhizome to normal flowering. However, in the CIR group, except the bud stage, there were no significant differences on DEG numbers among different tissues (Fig. 2c, the CIR group), which indicated that the response of *F. dibotrys* to the CIR treatment tend to be the whole plant.

### The different spatiotemporal regulations of the epicatechin biosynthetic pathway genes under normal flowering and the CIR treatment

According to our previous study in *F. dibotrys*, epicatechin is the product of flavonoid pathway and is synthesized from anthocyanin by the catalysis of ANR [17]. To uncover the spatiotemporal regulation of normal flowering and the CIR treatment on the biosynthesis of epicatechin, we analyzed the expression changes of all the key enzyme genes in the epicatechin biosynthesis pathway. For each group, taking the sample of the top part of the branch (T) as the control, we calculated whether there are any spatial expression changes in other tissues.

**Fig. 3:**
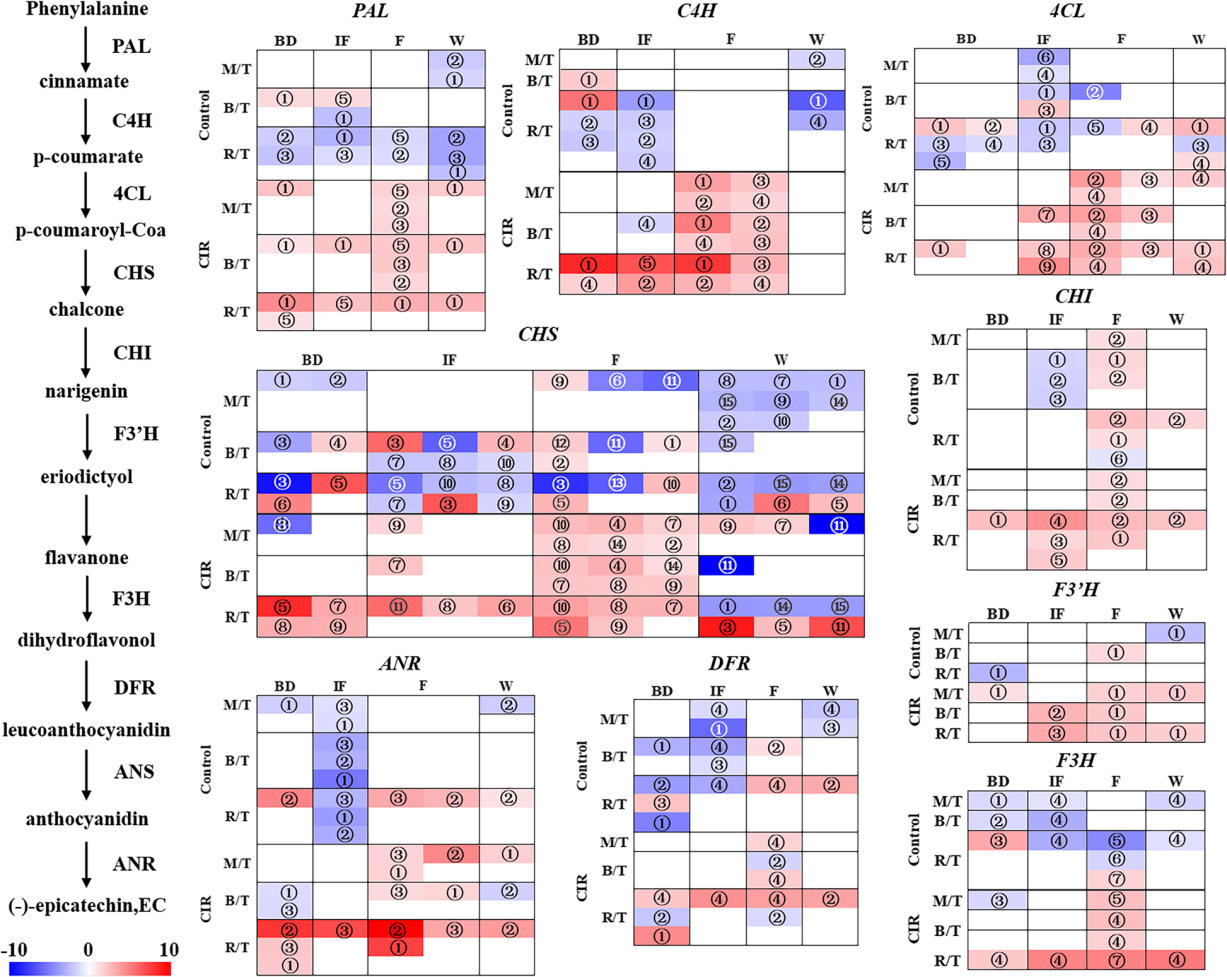
The spatiotemporal expression patterns of the key enzyme DEGs involved in the biosynthesis of epicatechin. The left pattern indicated the key enzyme genes involved in the epicatechin biosynthesis pathway in *F. dibotrys* according to Chen et al. (2016) [17]; the right pattern showed the spatiotemporal expression changes of the above genes. The abbreviations of the stages, groups and tissues were consistent with Fig. 1 and Fig. 2. For each group at each stage, the expression level of the sample T (the top part of the branch) was set as the control, thus the expression changes of the sample M/B/R were indicated as M/T, B/T and R/T. In the heatmap, the different members of each enzyme genes were randomly numbered from ①, which was indicated in the heatmaps

As shown in Fig. 3, except *ANS*, all the key enzyme genes of epicatechin biosynthetic pathway including *PAL*, *C4H*, *4CL*, *CHS*, *CHI*, *F3’H*, *F3H*, *DFR* and *ANR* were identified. All of them exhibited different spatial expression patterns in response to normal flowering and the CIR treatment. Under normal condition, all the key enzyme genes were down-regulated spatially at all stages, with the sharpest decline in the rhizomes. Although the expressions of *CHS*, *CHI* and *ANR* were improved at the flowering (F) stage, the increasement was 1 ~ 3 times lower than that of the CIR group (Supplementary Table S7).

On the contrary, under the CIR treatment, all the key enzyme genes including cinnamate 4-hydroxylase (*C4H*), *CHS*, *ANR*, *PAL* and flavanone 3-hydroxylase (*F3H*) were expressed highly during the whole process. In spatial dimension, *C4H*, *CHS* and *ANR* exhibited clearly gradient of up-regulations which increased 3 ~ 5 times from the top of branch to the rhizome, especially at the initial flowering (IF) and the flowering (F) stages. Therefore, in comparison with the widely repressions under normal flowering, the expressions of all the key enzyme genes involved in epicatechin biosynthetic pathway were significantly enhanced by the CIR treatment in both time (from bud stage to harvest) and spatial dimensions (from the top of branch to rhizome).

### Integrated analysis and co-expression analysis of the gene expression pattern and the epicatechin level

To verify the correlations between the gene expression pattern and the epicatechin level, the integrated analysis of LC–MS/MS and the rhizome transcriptome was further performed. Since the levels of epicatechin increased continuously during the whole process, while the expression patterns of most enzyme genes were upregulated until the flowering stage, no significant correlation was found between the two datasets. However, when we only focused on the first 3 stages, the expression patterns of 3 *PALs*, 2 *C4Hs*, 2 *4CLs*, 4 *CHSs* and 3 *ANRs* showed significant positive correlations with the level of epicatechin under both normal flowering and the CIR treatment (Table 1), which suggested the important roles of these *PALs*, *C4Hs*, *4CLs*, *CHSs* and *ANRs* in the biosynthesis of epicatechin.

**Table 1:** The integrated analysis of the LC–MS/MS and the transcriptome data at the first three stages

Family	Gene ID and the member number	The *Person* value of the correlation between the expression pattern and the changes on epicatechin level
*PAL*	Cluster-13504.27541, ②	0.516^a^
	Cluster-13504.27602, ③	0.575^a^
	Cluster-13504.20055, ⑤	0.718^b^
*C4H*	Cluster-13504.26574, ②	0.641^b^
	Cluster-13504.32177, ④	0.612^b^
*4CL*	Cluster-13504.22965, ①	0.535^a^
	Cluster-13504.25583, ④	0.830^b^
*CHS*	Cluster-13504.26998, ②	0.764^b^
	Cluster-13504.24227, ⑦	0.502^a^
	Cluster-13504.27150, ⑧	0.472^a^
	Cluster-13504.24016, ⑨	0.504^a^
*ANR*	Cluster-13504.29296, ①	0.747^b^
	Cluster-13504.6433, ②	0.620^b^
	Cluster-13504.28087, ③	0.520^a^

^a^ Indicated the correlation is significant at the 0.05 level

^b^ Indicated the correlation is significant at the 0.01 level

To identify the key TFs that potentially function in the biosynthesis of epicatechin, we also performed the co-expression analysis between all the differentially expressed TFs and the key enzyme genes in epicatechin biosynthetic pathway. Using the threshold of |r|> 0.8, a total of 93 TFs were identified to correlated with the epicatechin biosynthetic pathway genes. Among them, a total of 77, 10, 6, 5 and 5 TFs were related to *CHS*, *ANR*, *F3H*, *DFR* and *4CL*, respectively (Supplementary Table S8). The TFs that positively co-expressed with *ANR* included MYB (Cluster-13504.47067, Cluster-13504.9704), bHLH (Cluster-13504.13798), SNF2 (Cluster-13504.13828), SRS (Cluster-13504.13378), AUX/IAA (Cluster-13504.31854), HB-WOX (Cluster-13504.6744) and others (Cluster-13504.6848).

### The different spatiotemporal regulations of the flowering regulators under normal flowering and the CIR treatment

In *Arabidopsis*, it was revealed that, spatially, the expression levels of *SPL* decreased gradually from the top to the basal part of stem, while the flowering repressor *DELLA* could interfere with *SPL*’s transcriptional activity. With the involvement of some stress-responsive genes or TFs, *SPL* and *DELLA* could inhibit or promote the biosynthesis of anthocyanin, respectively [8–12]. Epicatechin is also the product of flavonoid pathway, so we further investigated whether the effects of flowering or the CIR treatment on the biosynthesis of epicatechin is mediated by the above genes or TFs.

**Fig. 4:**
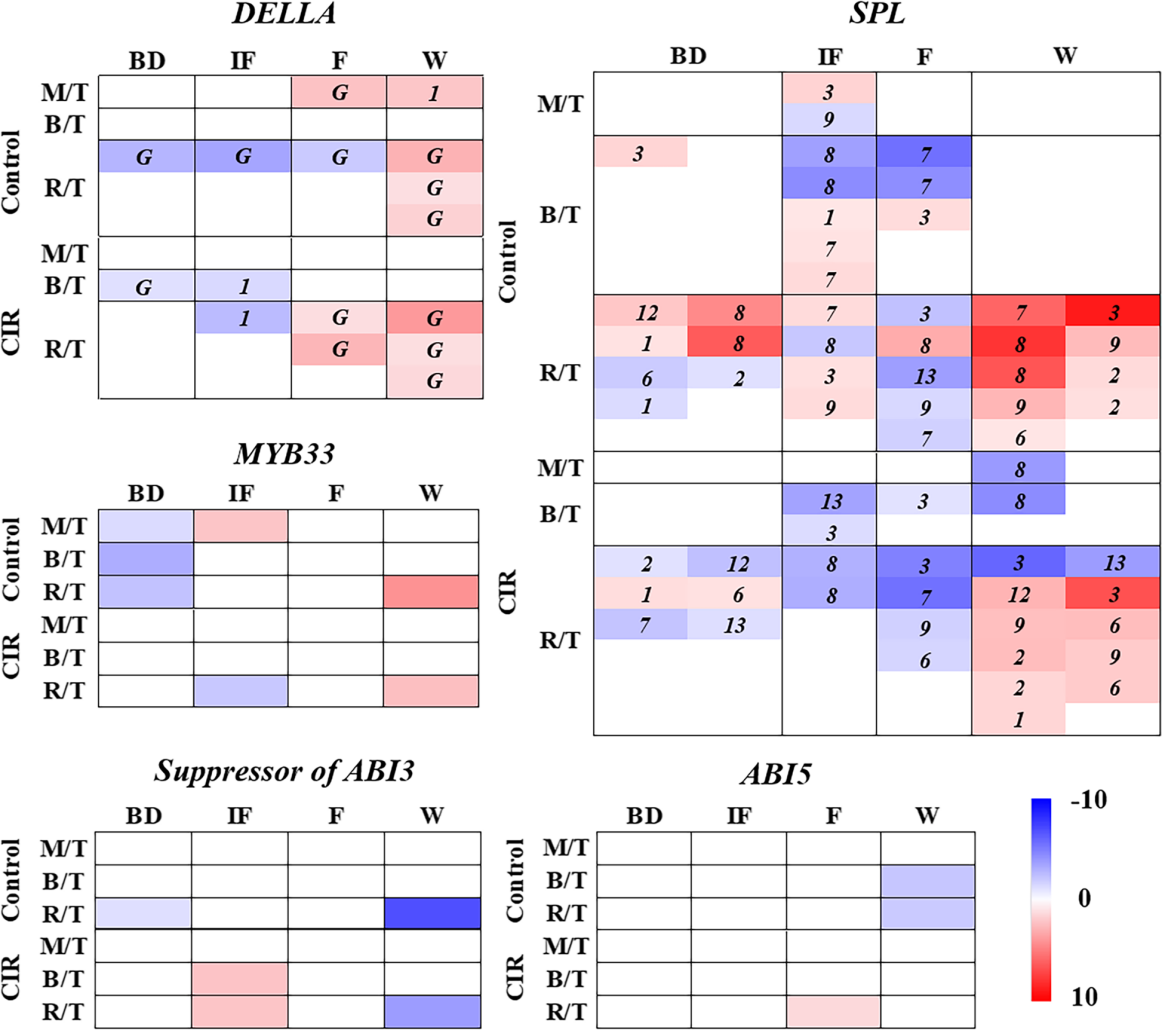
The spatiotemporal expression patterns of the DEGs involved in flowering and the biosynthesis of flavonoids. The abbreviations of the stages, groups and the tissues were consistent with Fig. 1 and Fig. 2. For each group at each stage, the expression levels of the sample T (the top part of branch) were set as the control, thus the expression changes of the sample M (the middle part of branch)/B (the basal part of branch)/R (the rhizome) were indicated as M/T, B/T and R/T. In the heatmap, *DELLA* and *SPL* were classified according to the homologous genes in *Arabidopsis*. *G*: *GAI*

**Fig. 5:**
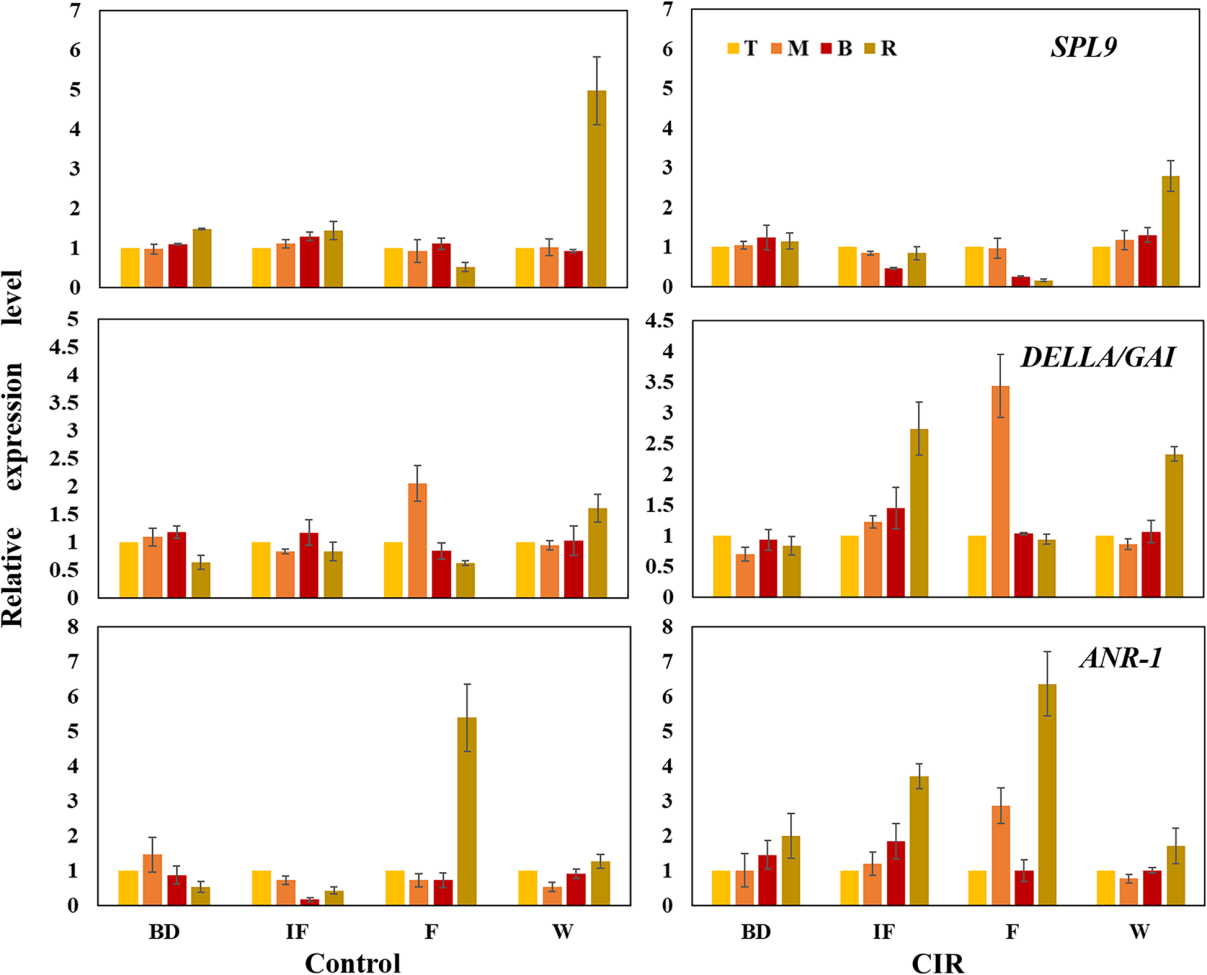
The qRT-PCR analysis of the spatiotemporal expression patterns of *SPL9*, *DELLA/GAI* and *ANR-1*. The abbreviations of the stages, groups and the tissues were consistent with Fig. 1 and Fig. 2. For each group at each stage, the expression level of the sample T (the top part of the branch) was set as the control. Three biological replications and three technical replications were conducted for each sample

For each group, the spatial expression changes were analyzed by the control of the sample T (the top part of branch). In total, we identified 26 differentially expressed *SPLs* belonging to *SPL1/2/3/6/7/8/9/12/13*, which were proved to promote phase change and accelerate flowering in *Arabidopsis* or switchgrass [25–27]. Most of them were up-regulated at the bud stage (BD) and the withering stage (W) and down-regulated at the initial flowering stage (IF) and the flowering stages (F), with the strongest response in the rhizome. In comparison with the normally bloomed plants at the same stage, the expression levels of *SPL2/3/8/9* were down-regulated by 2 ~ 3 times in the rhizomes under the CIR treatment (Fig. 4, Supplementary Table S7). The expression patterns of *SPL9* (*Cluster-13504.18693*) were also confirmed by qRT-PCR (Fig. 5).

Besides, four differentially expressed *DELLAs* belonging to *GAI* and *DELLA1* were identified. In time dimension, *DELLAs* were down-regulated at the early stages (BD and IF) and up-regulated at the late stages (IF and W). However, the spatial expression gradient of all *DELLAs* were not as obvious as that of *SPLs*. Under the CIR treatment, the expression levels of *DELLAs* only decreased by 1 ~ 2 times than that of the normally bloomed plants. No significant differential expressions were found for the *suppressor of ABI3*, *ABI5* and *MYB33* (Fig. 4, Supplementary Table S7). The expression patterns of *DELLA3* as well as *ANR* were confirmed by qRT-PCR (Fig. 5).

### Co-expression analysis and WGCNA of flowering regulators and epicatechin biosynthetic genes

**Fig. 6:**
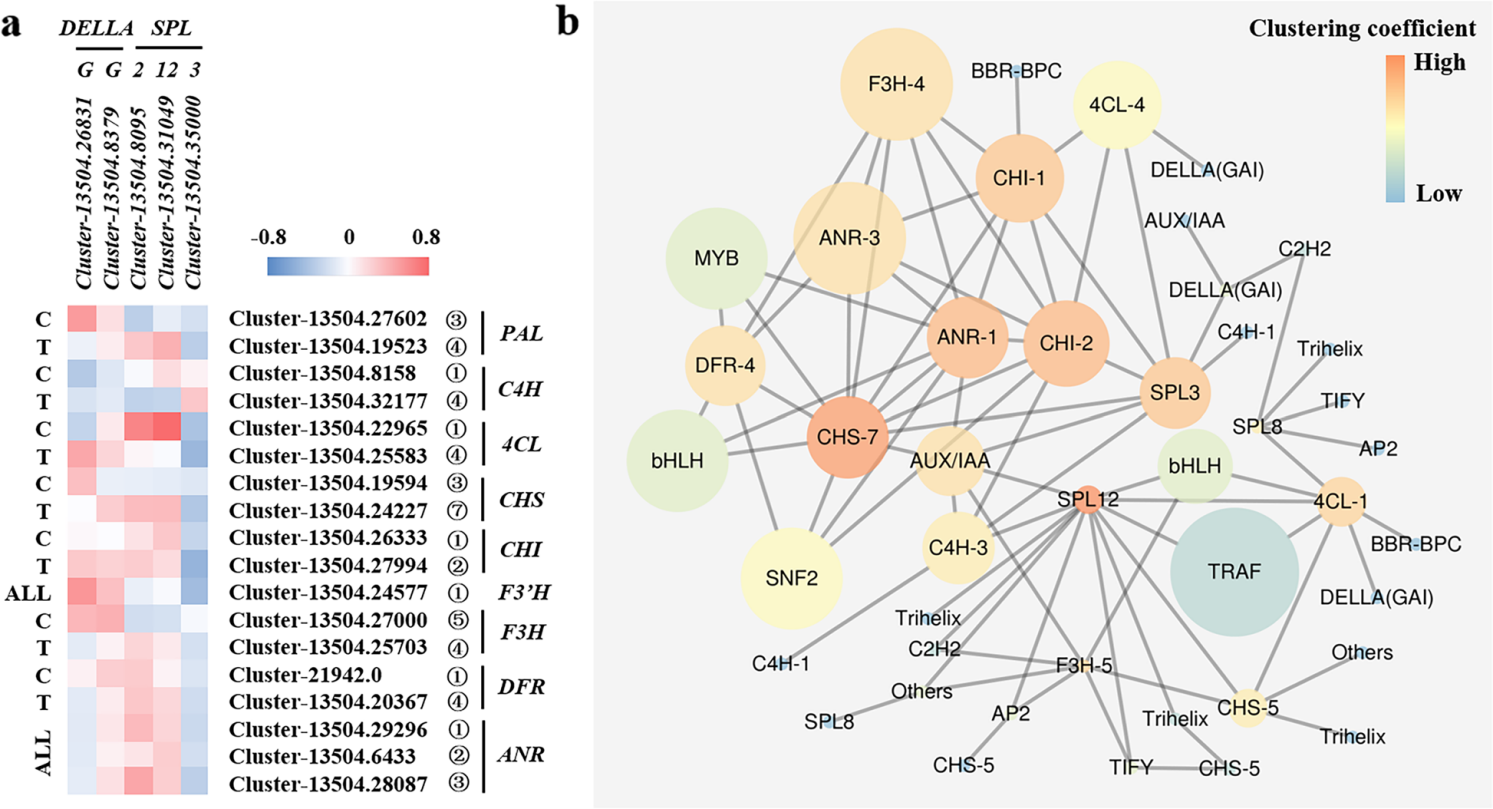
Co-expression analysis and WGCNA of the genes/TFs involved in epicatechin pathway and flowering regulation. **a** Co-expression analysis between the flowering process (the flowering activator *SPLs* and the flowering repressor *DELLAs*) and the biosynthesis of epicatechin. The heatmap was generated based on the *r* value. The genes or TFs that showed spatial differential expressions at more than three stages were selected for analysis. The naming rules was identical to that of Fig. 3 and Fig. 4. **b** Weighted gene co-expression network analysis (WGCNA) of the *SPLs*, *DELLAs* and the epicatechin biosynthetic pathway genes. Low value of clustering coefficient was indicated by dark blue. The naming rules was identical to that of Fig. 3 and Fig. 4

To investigate the correlations between normal flowering or the CIR treatment and the biosynthesis of epicatechin, co-expression analysis was performed between *DELLAs*, *SPLs* and the genes involved in epicatechin biosynthetic pathway. According to transcriptome profiles (Fig. 4), two representative members that showed the most significant spatial differential expressions in the normal flowering group and the CIR group were selected for each family, respectively. As shown in Fig. 6a, *SPL3*, which has been known to strongly expressed in response to floral induction compared with other members [4], were found to negatively co-expressed with almost all the enzyme genes in epicatechin pathway as expected. *SPL2/12* could negatively co-express with *PALs*, *CHSs*, *F3Hs* under normal flowering as expected, but positively co-expressed with those enzyme genes under the CIR treatment; opposite situations were found for *C4Hs* and *4CLs*; for *CHIs*, *DFRs* and *ANRs*, the two groups showed identical results. No significant regular patterns were found for *DELLAs*.

We further performed WGCNA based on all the genes or TFs included in correlation analysis (Supplementary Table S8) and co-expression analysis (Fig. 6a). Consistent with Fig. 3 and Fig. 6a, *SPLs* played a much more important role in regulating the biosynthesis of epicatechin than *DELLAs* under either normal flowering or the CIR treatment. *SPL3* was highly correlated with several enzyme genes that showed significant spatial expression changes in response to the CIR treatment, such as *4CL-4*, *CHI-1*, *CHS-7* and *C4H-4*. *SPL3* was also directly correlated with the *AUX/IAA*-involved auxin pathway; *SNF2*, *MYB* and *bHLH* were also engaged in the above regulations. While, *SPL8* and *SPL12* were mainly correlated with TFs, such as *TRAF* and *bHLH*, and some enzyme genes that mainly response to normal flowering, such as *F3H-5* and *4CL-1*.

## Discussion

### Correlation of flowering to the spatiotemporal gene regulations of *SPL-DELLA* and epicatechin biosynthetic pathway

In this study, we identified 26 *SPLs* belonging to *SPL1/2/3/6/7/8/9/12/13* in *F. dibotrys* (Fig. 4). With the cooperation of all the members, *SPLs* formed an expression gradient that increased from the top of branch to the rhizome under normal flowering, while the CIR treatment could eliminate or largely weaken this gradient (Fig. 2c, Fig. 4, Fig. 6a). These results extended the range of the spatial expression gradient of *SPLs* from aboveground to underground [12], and indicated the strong effects of the flowering process and the CIR treatments on the secondary metabolism in the roots/rhizomes of plants.

**Fig. 7:**
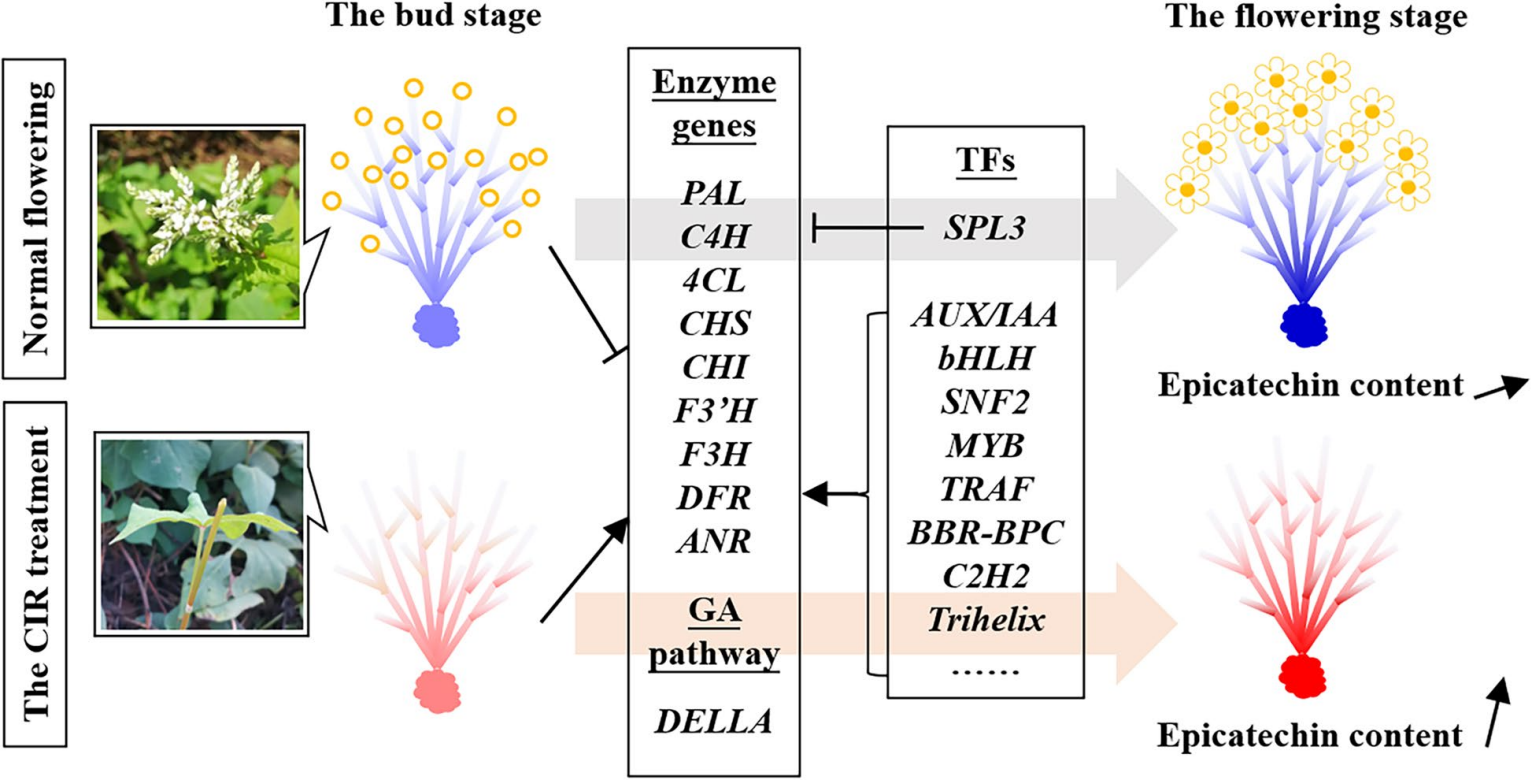
A model for the spatiotemporal regulations of the biosynthesis of epicatechin in *F. dibotrys*. The red and blue color of the plant indicated the up-regulation and down-regulation of the epicatechin biosynthetic pathway genes, respectively. The black arrows indicated the correlations according to the results of WGCNA in this study

Moreover, the spatiotemporal expression patterns of *SPLs* were converse to that of the epicatechin biosynthetic genes as expected (Fig. 3). However, considering the excessive number of members that involved, few *SPLs* could significantly co-express with the enzyme genes, which indicated that most of *SPLs* might involve in the regulation of epicatechin pathway only in particular stage or tissue. Besides *SPL9/10/13* which was reported to inhibit the biosynthesis of some flavonoids like anthocyanin [10, 12], we found that *SPL3* showed the most significant negative correlations with all the enzyme genes in epicatechin biosynthetic pathway (Fig. 6). In *Arabidopsis*, *SPL3* was proved to promote floral meristem identity transition [25]. In all, the above results might be an important reason why the biosynthesis of epicatechin is inhibited during normal flowering and enhanced after the CIR treatment (Fig. 7).

*DELLA* didn’t exhibit significant correlations with any enzyme genes of epicatechin biosynthetic pathway (Fig. 4, Fig. 6). We consider that although plant will bloom after the completion of phase change, there is still a lagging process of phase change in spatial dimension after flowering; *SPL* might still be the dominant regulator of this process, and further affects the biosynthesis of epicatechin, rather than *DELLA* or GA pathway. Consistently, in rice, it was revealed that the *GA3ox2* loss-of-function mutation didn’t affect the expression levels of miR156, which targets *SPLs* [3, 28].

Notably, at whole-plant level, we also firstly revealed the spatiotemporal expression patterns of all the key enzyme genes in the epicatechin biosynthetic pathway in response to normal flowering and the CIR treatment. Among the key enzyme genes, *CHS*, *CHI*, *4CL* and *C4H* could directly co-expressed with *SPL3*, which indicated that *SPL3* might mediate the effect of normal flowering and the CIR treatment on the biosynthesis of epicatechin in the rhizomes mainly through the negative regulations of the above enzyme genes. Moreover, it is also suggested that the levels of their products such as p-coumarate, chalcone or other flavonoids might also be improved by the CIR treatment. However, in *P. notoginseng*, the contents of flavonoids were decreased in roots after one-time inflorescence removal [15]. We considered the one-time treatment might not enough to trigger the *SPL*-dominated spatial phase change in plant. Therefore, it is indicated that the biosynthesis of flavonoids in the rhizome/root was highly correlated to the state of vegetative or reproductive growth of plant.

The stress responsive genes/TFs, such as *ABI5*, *ABI3* and *MYB33*, were proved involve in the interactions with *SPLs* and the accumulation of anthocyanin [8, 9]; while they didn’t show significant differential expression in this study (Fig. 4), which indicated that in *F. dibotrys*, the CIR treatment might not regulate the biosynthesis of epicatechin through abiotic stress pathways.

### Structural genes and transcription factors that involved in epicatechin biosynthesis

In this study, we only focused on the level of epicatechin under normal flowering and the CIR treatment (Fig. 1). On the one hand, only epicatechin has been listed as the bioactive compound of *F. dibotrys* in Chinese Pharmacopoeia; on the other hand, our quantified metabolome data showed that, the amount of epicatechin was dozens or even hundreds of times higher than that of the most detected precursors (Supplementary Table S2); moreover, epicatechin showed good stability during the 24 h stability test, and the modified products of epicatechin such as (-)-Epigallocatechin (EGC), (-)-Epicatechin gallate (ECG) or (-)-Epigallocatechin gallate (EGCG) cannot be detected because of the very limited contents (data not shown). These results confirmed that these products are not the main products in the rhizome of *F. dibotrys* [16, 29].

In this study, the epicatechin level was most positively correlated with the expressions of *ANRs*, followed by *CHSs* (Table 1), which was consistent with our previous study that the ectopically high expressions of *ANR* and *CHS* were contributed for the accumulation of epicatechin in another mutant line of *F. dibotrys* [17], and confirmed again the pivotal roles of *ANR* and *CHS* in the biosynthesis of epicatechin in *F. dibotrys*. *CHI* has also been reported to reduced level of anthocyanin by up-regulating *ANR* [30, 31]. However, in our integrated analysis, *CHI* didn’t show significant correlation with epicatechin level (Table 1). Instead, as shown by integrated analysis, co-expression analysis and WGCNA, we uncovered the potential important roles of *4CL* and *DFR* in the biosynthesis of epicatechin for the first time; the expression of *4CL* was highly correlated with *SPL* and *DELLA*, while *DFR* was only correlated with *ANR* (Table 1, Fig. 5, 6).

A total of 93 TFs including S*PL*, *MYB*, *bHLH*, *SNF2* were identified to be significantly correlated with *ANR*, *CHS* and *4CL*, respectively (Supplementary Table S8). According to WGCNA, some identified *SPLs* showed significant correlations with *CHS*, *CHI*, *4CL* and *C4H* (Fig. 6b), which was different with the previous studies that *SPL* could repress the biosynthesis of anthocyanin by prevent the expression of *F3’H* and *DFR* [12, 32]. Thus, it was indicated that the regulations of the flavonoid pathway genes by *SPL* might depend on metabolites. *AUX/IAAs* were found to only correlated with *ANR*, *C4H* and *SPL3* (Fig. 6b), which suggested the notable potential interactions between root growth and the biosynthesis of epicatechin, and provided new clue to our understanding of the importance of growth years in the quality control of medicinal plant. Other TFs such as *MYB*, *SNF2* and *bHLH* could also directly correlated with *ANR*, *CHS* and *CHI* (Fig. 6b). In many model and horticultural plants, *MYB* and *bHLH* were widely demonstrated to involved in the regulations of *ANR*, *LAR*, *FLS,* etc. [33–35]. *SWI/SNF* complex was known to mediate ATP-dependent chromatin remodelling to regulate gene expression; several subunits of *SWI/SNF* were demonstrated to involve in the control of flowering and potentially correlated with DNA methylation [36]. Other TFs, such as *BBR-BPC* and *Trihelix*, might be the novel regulators in the epicatechin biosynthetic pathway.

## Conclusions

In this study we found that the classic model of the spatial expression gradient of *SPL* appears to apply to flowering process in *F. dibotrys*. Moreover, the range of such spatial expression gradient could be extended from aboveground to underground, and further apply to all the key enzyme genes of epicatechin biosynthetic pathway in a reverse pattern. The CIR treatment could enhance the epicatechin level by sharply altering the spatiotemporal expression patterns of the above genes/TFs. *SPL3* appears to mediate the effect of normal flowering and the CIR treatment on the biosynthesis of epicatechin in the rhizomes mainly through the negative regulations of its key enzyme genes including *CHS*, *CHI*, and *C4H*. Other TFs such as *AUX/IAA*, *SNF2*, *MYB* and *bHLH* were potentially engaged in the biosynthesis of epicatechin. Taken together, this study provides new insights into the molecular mechanism of flowering-regulated secondary metabolism in the roots/rhizomes of medicinal plants or other crops.

## Methods

### Plant materials and treatments

The *F. dibotrys* used in this study was the new line separated from M_2_ generation of the γ-Co^60^ irradiated Jiangsu germplasm. This new line exhibited red leaves and was authenticated by Dr. Ailian Li of the Institute of Medicinal Plant Development (IMPLAD). It was reproduced asexually and had stable characters [17, 19].

The plants of both the control and the CIR groups were obtained by propagating from the one-year-old rhizome of this new line, a total of 30 plants were randomly selected for each group. All the plants were planted at April 16 of 2020 in the same plot of the experimental base of IMPLAD (lat. 39°47’ N, long. 116°25’ E, alt. 50 m).

Since August of 2020, we observed every day to see whether the flower bud appears. For the CIR group, as soon as the flower bud firstly appeared, the CIR treatment by removing the inflorescence with scissors was started. The treatment was carried out every day throughout the whole flowering period until the withering period, which continued two and a half months in total. All the plants in the CIR group were treated since August 21 (average temperature 22.5 ℃) and were sampled at different date: August 28 (average temperature 24.5 ℃) for the bud stage (BD), when the most branches are budding and the most buds are still green; October 7 (average temperature 15 ℃) for the initial flowering stage (IF), when the most buds are preparing to bloom (turns to white); October 20 (average temperature 12.5 ℃) for the full flowering stage (F), when the most buds bloom completely; and November 9 (average temperature 9.5 ℃) for the withering stage (W), when all the flowers withered and the rhizome was harvested. The inflorescence of the normal flowering group was saved.

For each stage, three plants from the control and the CIR groups were randomly selected, respectively. For each plant, the top part (T, the top of the branch, approximately 5 cm in length) of 3 branches were mixed as one sample; the middle part (M, at 1/2 of plant height, approximately 5 cm in length) and the basal part (B, approximately 5 cm in length) were sampled in the same way. The rhizome (R) of each plant was seen as one sample. For each rhizome sample, some part was used as the fresh sample for transcriptome sequencing, the other part was dried for the chemical analysis. In all, at each stage, there were 4 samples for each plant and 3 biological repeats for each group. A total of 96 fresh samples were used for transcriptome sequencing.

### Metabolomics analysis

The harvested rhizomes from the normal flowering group were used as materials. After freeze-drying, the rhizomes were grinded into power and ultrasonicly extracted; the supernatants were taken after centrifugation (12,000 rpm, 15 min) and filtration. The target compounds were separated by EXON LC System (SCIEX) UPLC. Mass spectrometry analysis was performed by SCIEX 6500 Qtrap with MRM mode. Peak detection and identification were conducted with the database constructed by ourselves [15].

### LC–MS/MS analysis of the contents of epicatechin

The reference substance of epicatechin was purchased from Beijing Jiachen Technology Co., Ltd. (Beijing, China) with purity ≥ 97% as determined by HPLC. HPLC-grade methanol and acetonitrile were purchased from Fisher (Emerson, IA, USA). Other reagents and chemicals were supplied by Sinopharm Chemical Reagent Beijing Co. Ltd (Shanghai, China). A MilliQ® purification system (Millipore, Billerica, MA, USA) was used to prepare deionized water for all experiments.

According to Qiao et al. [37], approximately 2 g of powder from each rhizome sample were accurately weighed and were extracted by methanol. LC–MS/ms system consisting of an Agilent 1260 Infinity HPLC system (Agilent Technologies, USA) and an AB SCIEX 4500 QTRAP MS system (AB SCIEX, Canada) were performed for sample analysis. An Agilent Poroshell 120 SB C18 column (100 mm × 2.1 mm, 2.7 µ m) was used for the HPLC separations, and the mobile phase for gradient elution consisted of (A) water (containing 0.1% formic acid) and (B) acetonitrile with the following gradient procedure: 0 ~ 4.0 min 10 ~ 25% B, 4.5 ~ 7.0 min 90% B and 7.1 ~ 10.0 min 10% B, with a flow rate of 0.30 mL/min. The injection volume was 2.0 µL. The column effluent was monitored by 4500 QTRAP MS system. The electrospray ionization (ESI) in the negative-ion mode and multiple reaction monitoring (MRM) scanning was employed for quantification. The related source settings and instrument parameters were listed in Table 2. Chromatographic workstation (v1.6.2) was used for the data analysis.

**Table 2:** MS parameters for detection of epicatechin

Analytes	Molecular Formula	t_R_ (min)	m/z Precursor	m/z Productions	DP (V)	CE (eV)
epicatechin	C_15_H_14_O_6_	6.07	288.8	122.9^(a)^/108.8	-80	-40
Source temperature (℃)			550			
Ionization voltage (V)			-4500			
Ion source (GS1) setting (psi)			50			
Ion source (GS2) setting (psi)			55			
Curtain gas setting (psi)			35			
Collision activation dissociation			Medium			
Dwell time (ms)			100			
Entrance potential (V)			-10			
Collision cell exit potential (V)			-15			

^a^ Product ion used for quantification. *Abbreviations*: *tR* Retention time, *DP* Declustering potential. *CE* Collision energy

### RNA isolation and transcriptome library construction

Total RNA of each sample was extracted using the RNeasy plant mini kit (Qiagen, Germany) according to the manufacturer’s instructions and was examined and quantified using a Nano Photometer spectrophotometer (IMPLEN, USA). A total amount of 1.5 μg of high-quality total RNA was used for transcriptome libraries construction, as described previously [38]. Then the libraries were sequenced using the Illumina HiSeq 2100 instrument (Novogene, China).

### Transcriptome assembly and annotation

After sequencing, the clean reads were obtained by removing reads containing adapter, reads containing N base and low-quality reads from raw data. The value of Q20, Q30 and GC content of the clean reads were calculated. Then, the clean reads were De novo assembled using Trinity with the default parameters [39]. Corset hierarchical clustering was used to remove redundant sequences, and the longest cluster sequences were obtained after Corset hierarchical clustering for subsequent analysis. We used BUSCO software to test the quality of the assembled TRINITY.fasta, unigene.fa and cluster.fasta files, and evaluated the accuracy and integrity of the assembled results. Subsequently, using BLAST, the longest cluster sequences were annotated by Nr, Nt, KO, SwissProt, Pfam, GO and KOG databases with E-value cutoff of 10–5, respectively.

### Differential expression analysis

Gene expression levels were calculated by FPKM (fragments per kilobase of transcript per million fragments mapped). Differential expression analysis of any two representative samples (three biological replicates per representative sample) was performed using the DESeq2 R package (v1.20.0). For each group in each stage, the expression level of the sample T (the top part of the branch) was set as the control; for KEGG analysis, we also took the normal flowering group as the control and analyzed the DEG enrhichment of the CIR group in each stage. The resulting *P*-values were adjusted using the Benjamini and Hochberg’s approach from controlling the false discovery rate. Genes with ana adjusted *P*-value < 0.05 and |log_2_FoldChange|> 1 were assigned as differentially expressed [40].

### GO and KEGG enrichment analysis of DEGs

GO enrichment analysis of DEGs was implemented by the clusterProfiler R package, in which gene length bias was corrected. GO terms with adjusted *P*-value < 0.05 were considered as significantly enriched DEGs. We also used cluster Profiler R package (v3.16) to test the statistical enrichment of DEGs assigned in KEGG pathways [41].

### Corelation analysis between the transcriptome data and metabolome

For each rhizome sample, the data of epicatechin contents and the data of expression levels (FPKM) of genes involved in the biosynthesis of epicatechin (showed differentially expression at 3 stages or more) were used for correlation analysis, the Person correlation coefficient (r) was calculated.

### Co-expression analysis and WGCNA

Among all the 96 samples, the expression levels (FPKM) of enzyme genes involved in the biosynthesis of epicatechin and TFs were calculated. The threshold of |r|> 0.6 was identified as significantly correlated.

Then, data processing was performed by using the WGCNA package in R-Studio 3.6.0 software. The average coverage of three biological replicates for 32 samples were used for analysis. An adjacency matrix was constructed using a soft threshold power of 12. The scale-free network was constructed using the blockwise Modules function, the procedure of module partition analysis and modules definition were according to Greenham et al. (2017) [42]. Based on the data of network node correlation profile of each module, the molecular interaction networks were visualized by Cytoscape software (v3.5) [43].

### Quantitative reverse transcription PCR analysis

Three DEGs were selected for qRT-PCR analysis by using the Light Cycler 96 system (Roche, Switzerland) and TransStart Top Green qPCR Super Mix (Aidlab, China). The specific primers for the genes were listed in Supplementary Table S1. The cycling conditions were as follows: 94 °C for 30 s, 45 cycles at 94 °C for 5 s, 55 °C for 15 s, and 72 °C for 10 s. Each sample has 3 biological replicates and 3 technical replicates. *Actin* was used as the reference gene [44]. The 2^−ΔΔCt^ method was used to analysis the relative expression levels [45].

## Supplementary Information

**Additional file 1: Supplementary Figure S1.** The GO enrichment analysis of the DEGs under normal flowering and the CIR treatment.

**Additional file 2: Table S1.** Primer information.

**Additional file 3: Table S2.** Metabonomic data of the precursors and metabolite of epicatechin.

**Additional file 4: Table S3.** Summary of sequencing quality.

**Additional file 5: Table S4.** Summary of transcript and unigene assembly data.

**Additional file 6: Table S5.** Unigene sequence homology search against the public databases.

**Additional file 7: Table S6.** The number of DEGs of each comparison.

**Additional file 8: Table S7.** Information of the DEGs involved in the biosynthesis of epicatechin and reproductive growth.

**Additional file 9. **

## Data Availability

Sequencing data for *Fagopyrum dibotrys* (D. Don) Hara was available on NCBI (accession number PRJNA782233).
