# Supplementary material for: The spatiotemporal regulations of epicatechin biosynthesis under normal flowering and the continuous inflorescence removal treatment in Fagopyrum dibotrys
Source: BMC Plant Biol. 2022 Jul 29;22:379. doi: 10.1186/s12870-022-03761-z (PMC9336051; doi:10.1186/s12870-022-03761-z)
Supplement: Supplementary file 1 — Additional file 1: Supplementary Figure S1. The GO enrichment analysis of the DEGs under normal flowering and the CIR treatment. [file 12870_2022_3761_MOESM1_ESM.docx]

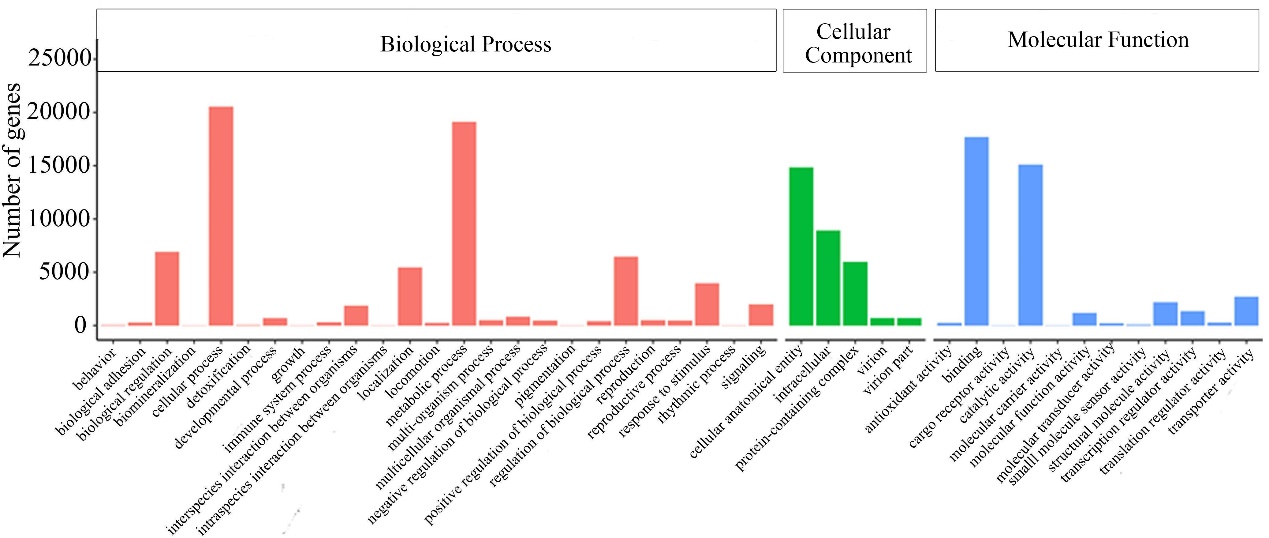


**Supplementary Figure S1** The GO enrichment analysis of the DEGs under normal flowering and the CIR treatment.

The normal flowering group was set as the control.
